# A New 3D Colon on a Chip to Decipher the Influence of Mechanical Forces on the Physiological Cellular Ecosystem

**DOI:** 10.1002/adhm.202505483

**Published:** 2026-01-24

**Authors:** Moencopi Bernheim‐Dennery, Lauriane Gérémie, Julie Brun, Lucas Chassatte, Giacomo Gropplero, Réda Bouras, Jieun Choo, Bertrand Cinquin, Alba Marcellan, Danijela Matic Vignjevic, Stéphanie Descroix

**Affiliations:** ^1^ UMR 168 CNRS‐Institut Curie IPGG PSL Research University Paris France; ^2^ UMR 144 CNRS‐Institut Curie PSL Research University Paris France; ^3^ UMR 7615 UPMC Sorbonne‐Université CNRS ESPCI Paris PSL Research University Paris France

**Keywords:** collagen, colon‐on‐chip, fibroblasts, organoids, PEGDA, peristalsis

## Abstract

The gut epithelium ensures nutrient absorption and barrier protection, functions tightly linked to its 3D architecture and dynamic mechanical activity. To dissect how mechanical forces influence intestinal physiology, we developed a stretchable 3D colon‐on‐chip that integrates tunable topography, stiffness and peristalsis‐like motion within a physiologically relevant microenvironment. The model employs 3D scaffolds composed of either pure collagen I or mechanically reinforced interpenetrated network (IPN) made of collagen I and PEGDA. Tensile tests mimicking peristalsis revealed that both hydrogels soften upon stretching, with the IPN maintaining higher stiffness than pure collagen. Using this platform, we applied cyclic stretching for 24 to 72 h to co‐cultures of stromal and epithelial cells, and systematically assessed the contributions of stiffness, curvature and shear stress. We found that the stretching was a dominant factor governing epithelial behavior, markedly enhancing proliferation and apicobasal polarization without altering differentiation. Altogether, this work introduces a next‐generation colon‐on‐chip that unites mechanical control and biological complexity, providing a powerful tool to unravel how physical cues orchestrate intestinal homeostasis and paving the way for modeling disease states such as colorectal cancer and inflammation.

## Graphical abstract

To dissect how mechanical forces influence intestinal physiology, we developed a stretchable 3D colon‐on‐chip that integrates tunable topography, stiffness and peristalsis‐like motion within a physiologically relevant microenvironment. We showed that stretching is a dominant factor governing epithelial behavior, markedly enhancing proliferation and apicobasal polarization without altering differentiation. This next‐generation colon‐on‐chip unites for the first time mechanical control and biological complexity.

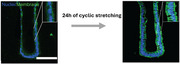

## 1 Introduction

The large intestine (colon) has a distinctive architecture composed of invaginations known as crypts, lined with a simple epithelium that is continuously renewed by stem cells residing at the crypt base. As cells migrate upward, they progressively differentiate [1]. The stem cell niche is maintained by surrounding fibroblasts within a collagen I–rich stroma [2, 3]. In addition to its highly organized 3D structure and spatial cell segregation, the colon epithelium is constantly exposed to mechanical forces. Shear stress arises from fecal flow, while to transport feces through the large intestine, longitudinal and circular smooth muscles undergo coordinated cycles of contractions [4]. These forces present are indispensable for normal colon function and profoundly influence the intestinal ecosystem under both physiological and pathological conditions [5]. Yet, dissecting their effects in vivo remains challenging due to limited control of mechanical inputs.

Microphysiological systems offer a way to reproduce organ features in vitro with precise control of the cellular environment [6, 7, 8, 9]. Two main strategies have been developed for the gut.

The first approach focuses on reproducing the 3D architecture of the gut and spatial organization of the epithelium. Epithelial cells are cultured on a 3D scaffold made of biomaterials such as collagen type I [8, 10], silk [11, 12], or synthetic hydrogels with poly(lactic‐co‐glycolic acid) (PLGA) [13, 14] or polyethylene glycol diacrylate (PEGDA) [15, 16]. While collagen offers physiological relevance, mechanical reinforcement often requires cytotoxic crosslinkers that limit stromal cell inclusion [17]. Only a few models enable the co‐culture of epithelial cells with other stromal cells in a 3D gut‐on‐chip setting. Among them, we previously developed a cytocompatible threose‐reticulated collagen gut‐on‐chip that supports epithelial–fibroblast interactions under shear stress within a high–aspect ratio structure [6]. More recently, M. Nikolaev et al. engineered perfusable tube‐shaped epithelia [18]. These models allow exploration of geometric and cellular compartmentalization effects but do not replicate physiological mechanical stimulation.

The second approach, pioneered by Ingber's group, focuses on mimicking intestinal mechanics. In this model, different cell types are cultured on opposite sides of a 2D porous ECM‐coated membrane subjected to shear stress and cyclic stretching [19, 20]. This configuration has revealed how mechanical cues shape epithelial, immune, and microbial interactions [7, 21, 22]. However, it lacks a crypt‐like 3D architecture, and the lamina propria with diverse cells and biochemical signals crucial for intestinal homeostasis.

Here, we present a novel colon‐on‐chip that integrates both 3D colon architecture and peristaltic motion, enabling the disentangling of mechanical force impacts on the colon ecosystem. To achieve this, we fabricated 3D scaffolds from either pure collagen or an interpenetrating collagen‐PEGDA hydrogel (IPN), where PEGDA enhances mechanical stability without compromising cytocompatibility. These hydrogels were molded into a stretchable chip reproducing physiological geometry and deformation. Mechanical tests showed that, although both matrices softened under stretching, the IPN retained higher stiffness even when fibroblasts were embedded. Finally, we used this versatile system to apply cyclic stretching to stromal and epithelial cells under varying scaffold stiffness, curvature, and shear stress. Stretching emerged as the dominant regulator of epithelial behavior, enhancing proliferation and apico‐basal polarization within the 3D colon‐like architecture.

## 2 Material and Methods

### 2.1 Material

Matrigel was purchased from Corning. Phosphate Buffered Saline (PBS, Sigma–Aldrich, Cat. No. D8537), DMEM glutamax (Thermo Fisher Scientific), Cat. No. 10566016), DMEM F12 (Thermo Fisher Scientific, Cat. No. 10565018), FBS (Thermo Fisher Scientific, Cat. No. A525670), ITS‐A (Thermo Fisher Scientific, Cat. No. 51300044), anti‐anti (ITS‐A (Thermo Fisher Scientific, Cat. No. 51300044), gentamicin (Thermo Fisher Scientific, Cat. No. 15710064), B27 (Thermo Fisher Scientific, Cat. No. 17504044), N2 (Thermo Fisher Scientific Cat. No. 17502048), LIVE/DEAD Fixable Blue Dead Cell Stain Kit, for UV excitation (Thermo Fisher Scientific, Cat. No. L23105) and AlamarBlue Cell Viability Reagent (Thermo Fisher Scientific, Cat. No. A50101), Laminin (Thermo Fisher Scientific, Cat. No. 23017015), High‐Capacity cDNA Reverse Transcription Kit (Thermo Fisher Scientific, Cat. No. 10400745), SYBR Green PCR Master Mix and Tryple from Thermofisher (Thermo Fisher Scientific, Cat. No. 10658255). PEGDA (Sigma–Aldrich Cat. No. 701971‐1G), LAP (Sigma–Aldrich, Cat. No. 900889‐5G), glutaraldehyde (Sigma–Aldrich, Cat. No. 340855‐25ML), Bovine Serum Albumin (BSA) (Sigma–Aldrich, Cat. No. A2153‐100G), 3‐aminopropyltriethoxysilane (APTES) (Sigma–Aldrich), Cat. No. 440140‐100ML) and Triton X‐100 (Sigma–Aldrich), Cat. No. X100‐100ML). Noggin (R&D Systems, Cat. No. 250‐38), EGF (R&D Systems, Cat. No. 315‐09), mbFGF (Peprotech). R‐spondin (R&D Systems, Cat. No. 3474‐RS‐250). Ecoflex 00–30 (Smooth‐On), Cat. No. EF0030pint. PDMS from (Neyco), Cat. No. DC184‐1.1. UV lamp (Omnicure, Lumen Dynamics, AC450‐365), CellScraper (Falcon, Cat. No. 353086), NucleoSpin RNA Kit (Macherey‐Nagel, Cat. No. 740955.50), Acetic acid (VWR, Cat. No. 20104.298).

### 2.2 Cells and Organoids

Primary mouse fibroblasts were obtained from colon tissue of aSMA‐CreERT2 mTmG flox mice. Briefly, the colon was isolated and cleaned with cold sterile PBS with 2% (v/v) Antibiotic‐antimycotic. The tissue was sliced into small pieces and they were placed on 11 kPa polyacrylamide gel coated with 100 µg/ml of collagen I. DMEM Glutamax supplemented with 10% (v/v) FBS, 1% (v/v) Insulin‐Transferrin‐Selenium, 2% (v/v) Antibiotic‐Antimycotic, 4 µg/mL ciprofloxacin and 12.5 µg/mL metronidazole was added very carefully. The medium was changed every 3 days until fibroblast emerged from the tissue. Cells were immortalized by retroviral infection of SV40 large T‐antigen. Fibroblasts were used at passage 15 to 28. Fibroblasts express membrane Tomato (red) and αSMA positive cells (including myofibroblasts) express membrane GFP. mTmG organoids or Lgr5/mTmG organoids were generated as previously described [6]. Briefly, the small intestine was isolated and cleared from its inner materials with a syringe filled with a cleaning solution (2% (v/v) antibiotic‐antimycotic, 1% (v/v) gentamicin in cold sterile PBS). Then, it was opened longitudinally, cut into 3 cm long pieces, and incubated in the cleaning solution with constant shaking at 4°C for 15 min twice. The tissue was then transferred in a cleaning solution supplemented with 10 mm Ethylendiacetic acid (EDTA) for 10 min at 4°C. The whole tissue was transferred again in the cleaning solution and vortexed for 2 min, 3 times by changing the cleaning solution between each vortex cycle. Then, the last two steps were repeated with an incubation in the EDTA solution of 5 min and vortex cycles of 3 min. The supernatant was filtered using a 70 µm strainer before spinning at 300 g for 4 min. The pellet was resuspended in a 1:1 ratio of ENR (DMEM F12 supplemented with EGF (Epidermal Growth Factor) 20 ng/mL, FGF (Fibroblast growth Factor)10 ng/mL, Noggin 100 ng/mL, R‐Spondin 500 ng/mL, B27 without serum 1X, N2 1X, anti‐anti 2X) + Matrigel. 50 µL drops were plated in 24 well plates and were allowed to polymerize for 30 min in a 5% CO_2_ humidified air incubator. After polymerization, 350 µL of ENR was added per well. Organoids were used up to passage 10.

### 2.3 IPN Hydrogel Fabrication

Collagen I was extracted from rat tails as described previously [23]. For the experiment, it is neutralized on ice with 1 M NaOH, 10X PBS and with fibroblasts (DMEM glutamax, 10% FBS, 1% ITS, 1% anti‐anti) medium. The collagen solution is prepared to reach a final concentration of 10 mg/mL. Collagen was mixed with 1.2.10^6^ fibroblasts/ml deposited into the desired support: well‐plates for cell viability assays, PDMS mold of 10 × 20 × 2 mm for mechanical tests and chip for stretching experiments, and let polymerize for 1 h at 37°C and 5% CO_2_. Media was added to chambers and cells were allowed to recover for 1d at 37°C and 5% CO_2_. PEGDA containing solution was prepared by mixing 11.7% w/v PEGDA 6.0 kDa and 0.2% w/v LAP (Lithium phenyl‐2,4,6‐trimethylbenzoylphosphinate) photoinitiator, the solution was sonicated for 30 min in a protected light environment to help the photoinitiator and the PEGDA solubilize. The culture medium was replaced by the PEGDA solution and incubated at 37°C, 5% CO_2_. After 2 h, PEGDA solution was removed, quickly rinsed with DMEM glutamax without phenol red, and polymerized for 6 min under 365 nm UV light at ≈10 mW/cm^2^. The resulting IPN gel was rinsed with cell culture medium for 30 min 3 times in the incubator.

### 2.4 Cell Viability Assay

The cell viability was evaluated 1, 7 and 14 days post‐fabrication of the IPN or control samples of collagen at 10 mg/mL. Live/Dead Cell Imaging Kit and AlamarBlue assay were used to evaluate viability of fibroblasts and organoids, respectively using protocols provided by the respective suppliers. For fibroblasts viability assay, fibroblasts are mixed with the collagen prepolymer solution prior to on‐chip injection and molding (with cylindrical shape), followed by the polymerization of collagen and the photopolymerization of PEGDA in the case of IPN hydrogel. The fibroblasts were seeded in collagen at 1.2 × 10^6^ cell/mL, which represents around 0.36 × 10^6^ fibroblasts per chip. For the organoid's viability experiments, fibroblasts were intentionally excluded. Since viability was assessed using a metabolic assay, the presence of fibroblasts would contribute to the measured signal and confounded the interpretation of organoid viability. Therefore, these assays were performed using organoids alone. For organoid experiments, the organoids were extracted from Matrigel after seven days in culture by scratching the Matrigel. Afterward, the matrix was pipetted up and down in ENR medium three to four times through a 200 µL pipette tip, then ten times through a 20 µL pipette tip in order to break them and obtain smaller cystic like organoids of about 100 µm in diameter. The organoids were subsequently centrifuged at 1400 rpm for 3 min. The pellet made of organoids was resuspended in ENR medium. 50 µL of two wells of a twenty‐four well plate‐worth of organoids in ENR solution were seeded on laminin‐coated collagen scaffolds and left for at least four hours in the incubator before the chips were put back in medium. Experiments were repeated at least twice per condition with two samples for each time point. Fluorescence images were acquired from 3 to 9 randomly selected regions per sample using a confocal microscope equipped with a 25X objective. With Alamar Blue, the viability was estimated using the following equation:

$$\frac{\left(\mathrm{average}\;\mathrm{RFU}\;\mathrm{value}\;\mathrm{of}\;\mathrm{the}\;\mathrm{experimental}\;\mathrm{control}-\mathrm{average}\;\mathrm{RFU}\;\mathrm{value}\;\mathrm{of}\;\mathrm{the}\;\mathrm{negative}\;\mathrm{control}\right)}{\left(\mathrm{average}\;\mathrm{RFU}\;\mathrm{value}\;\mathrm{of}\;\mathrm{the}\;\mathrm{positive}\;\mathrm{control}\;\mathrm{reduced}\;\mathrm{to}\;100\%-\mathrm{average}\;\mathrm{RFU}\;\mathrm{value}\;\mathrm{of}\;\mathrm{the}\;\mathrm{negative}\;\mathrm{control}\right)}\times 100$$

### 2.5 Mechanical Tests

Tensile tests until rupture and cycling experiments were performed on an Instron device, with sample holder especially designed for gel testing, using a home‐made chamber that enables to perform mechanical testing under controlled environment, i.e. in DMEM glutamax immersion at room temperature (RT). As mentioned above, IPN or control collagen at 10 mg/mL hydrogels, with or without embedded fibroblasts (1.2 × 10^6^ cells/ml), were deposited in a 10 × 20 × 2 mm PDMS mold and incubated 1 h at 37°C, 5% CO_2_. Nominal stress and strain were defined as follow: s = F/S_0_ and e = DL/L_0_, with S_0_ the initial cross‐section and L_0_ the initial length. Three to six independent samples were used to characterize the mechanical properties of each condition. The sample was placed between velcro gripping jaws with a tightening torque of 5cN.m to prevent slipping during the experiment and fixed on a 10N force cell. Conditions of tightening were optimized by performing tensile tests without environmental chamber and using a video extensometer, to ensure perfect agreement between local strain and asked strain. For monotonic tensile tests until rupture, the strain rate was set the same for each sample at 0.1s^−1^, i.e. in a way to correspond to a frequency of 0.1 Hz. The stress–strain curve was plotted for each measurement and the tensile modulus (E) was extracted from the linear region for each condition. For the cycling experiments, we characterized the response of the hydrogels under cyclic uniaxial loading over a long‐time experiment (8000 cycles) for a constant maximal elongation of 8% and frequency of 0.1 Hz, and the evolution of the tensile modulus (E) over cycles was followed.

### 2.6 Swelling

Swelling measurements were performed on three to six independent IPN and collagen samples. The samples were let to swell in DMEM Glutamax medium during 24 h after fabrication. The medium was then removed and the samples gently blotted before to record the swollen weight (Ws). Finally, the samples were placed in an oven at 70°C for 48 h to dry them before reweighing (Wd). We were then able to determine the final concentration of both collagen I and PEGDA within the final hydrogel. As expected, the collagen I is at 10 mg/mL, while within the IPN the final PEGDA concentration is about 6.3 ± 4.1 wt.%.

### 2.7 Chip Fabrication and 3D Molding

Microstructuring a colon on chip requires several steps as shown in Figure w3. First two 3D‐printed mold were fabricated. The bottom part was composed of a flat surface of 66 × 38 × 4 mm, delimited by walls of 3 mm height and a central square of 12 × 12 × 2 mm to mold a 2 mm deep pool that will later accommodate the 3D colon scaffold. The second part, the top one, had the same dimensions as the bottom part but contained 4 pillars of 3 mm height and 8 mm diameter to allow the formation of holes for plugging the chips on the stretcher. 3 mL of PDMS (Sylgard 184, Dow Corning) with a prepolymer/curing agent at 1:10 (v/v) were poured in the bottom part at a ratio 20:1 (w/w) with the curing agent. Then the top part of the 3D printed mold is positioned on the bottom part to imprint structures of the two parts of the molds on the PDMS structure. The PDMS chamber was next cured at 70°C for 2 h resulting in flat chips with the exact same dimensions: a 1 mm thick down membrane in the chamber and 7 mm thick walls, compatible with the stretcher reported previously [24]. To permit the collagen grafting to the PDMS chamber, the chips were plasma (plasma cleaner, Harrick) treated for 30 s followed by a treatment with 10% v/v of 3‐aminopropyltriethoxysilane (APTES) for 30 min at RT be under sterile conditions. After three washings with 70% ethanol, a solution of 2.5% v/v glutaraldehyde was added for 30 min and then washed again five times in water before being incubated at least overnight at 4°C. For the experiments, as detailed previously for small intestine6 [6], we prepared a second PDMS mold used a of the 3D crypt mold to further mold collagen. This PDMS stamp is fabricated as previously mentioned, using two distinct 3D printed molds. The first one replicates cylindrical crypts, spaced 125 µm apart: 250 µm in depth and 150 µm in diameter. The second mold features larger, cone‐like crypts: 250 µm deep and widening from 50 µm at the bottom of the crypt to 250 µm at the crypt opening. The crypt dimensions in the device are slightly larger than in vivo due to 3D printing limitations in mold fabrication. Two holes were made using punchers, one of 2 mm diameter for the injection and one of 0.75 mm for the outlet, at the edges of the stamp before plasma treating the 3D structure. Then, in sterile conditions, both parts (the chamber of the stretchable chip and the 3D PDMS stamp) were assembled with clips for collagen with fibroblasts injection. Fibroblasts are mixed with the collagen prepolymer solution prior to on‐chip injection and molding for all conditions except for the viability assays as mentioned above. This injection was performed through a Tygon tube using a conventional 1 mL syringe topped with a cut pipette tip. Once, the collagen filled the chip, it was let to polymerize for 1 h at 37°C, 5% CO_2_. For IPN gel, the addition of PEGDA and the photopolymerization was performed as described above. Finally, the unmolding step was performed delicately in a PBS bath supplemented with anti‐anti, and the chips were placed in a Petri dish with the corresponding culture medium.

### 2.8 The Stretcher System

The stretcher system comprises a linear servo (L12‐30‐210‐6‐R, Actuonix) connected to an ATmega328 microcontroller (Arduino Nano). The servo is directly powered by the Arduino's 5 V pin, the commands are sent from a computer using the Arduino's serial interface by an open‐source protocol (https://github.com/araffin/arduino‐robust‐serial/tree/master). The PDMS chips are attached using two 3D printed parts (resist: RG35B, BASF; printer: 028J+ HR, DWS): one at head of the servo and the other on the breadboard (MSB30/M, Thorlabs). PDMS substrates were fabricated using PDMS 10:1 (monomer/crosslinking agent) poured in a metal mold and incubated at 90°C for 2 h.

### 2.9 Organoids Seeding and Stretching

The 3D scaffold was incubated with 0.02 mg/mL laminin LN1 (Sigma) diluted in fibroblast medium for 1 h at 37°C, 5% CO_2_. The laminin solution was rinsed prior to organoids seeding. Prior to their seeding, the organoids were extracted from Matrigel and flushed several times through a 10 mL pipette mounted with a 200 and a 10 µL tip without filter to get small cystic organoid structures of about 100 µm in diameter. Then, the organoids were centrifuged at 1400 rpm for 3 min and the pellet was resuspended in ENR medium (DMEM F12 Glutamax supplemented with 20 ng/mL EGF, 10 ng/mL FGF, 100 ng/mL Noggin, 500 ng/mL R‐spondin, 1 × B27, 1 × N2). A drop of 50 µL of organoid solution was seeded on each scaffold. The organoids were left to adhere for 3 h at 37°C, 5% CO_2_ before being placed in a Petri dish filled with ENR medium and incubated for 4 days before stretching the chip. As the chip was designed flat, an Ecoflex reservoir, autoclaved before its use, was made to fit on the stretcher, adapted from Noor A. Al‐Maslamani et al. paper [24], while still allowing the chips to be plugged. ENR medium was added in a way to fill the reservoir and immerse the chip before starting the cyclic stretching for 1d at a deformation of 8% and frequency of 0.1 Hz.

### 2.10 Immunostaining

The chips were fixed in 4% PFA in PBS (v/v) for 30 min at RT and washed 3 times for 15 min with PBS. The cells were permeabilized with 0.3% Triton X‐100 (v/v) for 30 min at RT and rinsed 3 times for 30 min with PBS‐BSA 2% at RT. Primary antibodies against Ki67 (rabbit monoclonal, Abcam, Cat. No. ab16667), NaK‐ATPase (rabbit AF488 monoclonal, Abcam, Cat. No. ab197713), Fibronectin (rabbit monoclonal, Sigma, Cat. No. F3648), MUC2 (rabbit polyclonal, Santa Cruz Biotechnology, Cat. No. sc‐15334), LFABP (rabbit polyclonal, Santa Cruz Biotechnology, Cat. No. sc‐50380), anti GFP (rabbit polyclonal, Cell Signaling Technology, Cat. No. 2555) were applied on the cells and incubated overnight at RT. The chips were then rinsed 3 times for 30 min with PBS‐BSA 2% at RT. The chips were next incubated with the appropriate Alexa Fluor secondary antibody solution for 2 h at room temperature in the dark and then rinsed 3 times with PBS for 30 min at RT. The chips were stored at 4°C. They are finally embedded in Agarose 4% (Invitrogen) diluted in PBS before being cut using a vibratome (Leica) to make longitudinal slices of 400 µm. These latter are mounted in between a microscopy slide and a cover slip (VWR) using the Aqua‐Poly/Mount (Polysciences) mounting medium.

### 2.11 Fibronectin Quantification

To quantify the fibronectin signal, nuclei were segmented from the Hoechst channel using an IsoData threshold to create a binary mask. This mask was applied to the raw Hoechst data to calculate the mean intensity of the signal (excluding background) for every slice in the Z‐stack. Separately, the fibronectin signal was segmented and masked using a similar approach. The fibronectin signal in each slice was then normalized by dividing it by the corresponding slice‐specific Hoechst mean intensity. The average intensity per slice was next calculated from these normalized images, excluding the first and last three slices of each stack to minimize edge effects. Finally, the average intensity for each image was normalized by the number of cells counted manually.

### 2.12 RTqPCR

Epithelial cells were detached from the scaffold using Tryple, helped by scratching the surface with a CellScraper. RNA from the colon‐on‐chip was extracted using the NucleoSpin RNA Kit followed by the cDNA synthesis with High‐Capacity cDNA Reverse Transcription Kit. The qPCR was performed using SYBR Green PCR Master Mix (Fw: aggatccatctgtcctttggt and Rv: ttcagctgccttcttgttcc for Alpi, Fw: cacgagacccaggaagtacag and Rv: gcaaagccactaactgcttgt for MUC2, Fw: aacttcaagacctggctctcc and Rv: ctcaaagctgctgtgttgct for ChgA, Fw: gttgctgaaccagagccttc and Rv: ttggggtgtcacgagagg for Gfi1b, Fw: cttcactcggtgcagtgct and Rv: gatcagccagctaccaaatagg for Lgr5, Fw: cct cct cag acc gct ttt t and Rv: aac ctg gtt cat cat cgc taa hypoxanthine‐guanine phosphoribosyltransferase (HPRT)), and run on a QuantStudio3 Flex Real‐Time PCR System. Results were normalized relative to HPRT housekeeping gene expression.

### 2.13 COMSOL Simulation

We used the COMSOL Multiphysics software (version 6.2, COMSOL AB, Stockholm, Sweden) to model fluid flow at the interface between the fluid and crypt structures. The simulations were conducted using a 2D model, which included three rectangular crypts. Each crypt had a width of 150 µm, a depth of 250 µm, and the spacing between crypts was 100 µm. They were positioned beneath a laminar flow of water through a channel that was 500 µm in height and 1 mm in length. The inlet and outlet pressures were set to 6 mPa and 0 Pa, respectively. The computational mesh employed was the default fine physics‐controlled mesh provided by COMSOL. Results were visualized by plotting line graphs along three lines, each extending from the top center to the bottom center of the crypts.

### 2.14 Animals

Animal care and use for this study were performed in accordance with the recommendations of the European Community (2010/63/UE) for the care and use of laboratory animals (facility license # A1‐0056).

### 2.15 Imaging

3D scaffolds slices were imaged using a Leica DMi8 inverted SP8 confocal microscope equipped with a HC PL APO 40x/1,10 W CORR CS2 objective and 405, 491, 561 and 634 nm lasers controlled with the LasX software. Z stacks were acquired every 1 µm (40 × W) with a resolution of 1024×1024 pixels and images were processed using ImageJ software.

### 2.16 Quantifications and Statistics

Quantification of the proliferative cells was performed using the MIC‐MAQ ImageJ plugin (https://github.com/MultimodalImagingCenter/MIC‐MAQ). For the quantification of the epithelial polarization, an ImageJ Macro has been written. The middle plane representative of the longitudinal crypt is segmented. The distance map and the skeleton are coupled to extract the distance of each pixel to the outer point of the geometry given the height of the crypt in the apicobasal direction in each pixel. The average height over a whole crypt is evaluated and the total average height over all crypts is then plotted. All experiments were performed in 2–3 independent experiments on 2–3 chips per condition. Statistical analysis and graphic representations were performed using Graph Pad Prism software.

## 3 Results

### 3.1 Optimization of the Collagen/PEGDA IPN Synthesis for Long‐Term Survival of Intestinal Cells

To dissect the role of mechanical forces in the colonic ecosystem, we first established a stretchable 3D colon‐on‐chip model with tunable topography and stiffness. This system relies on 3D scaffolds made either from pure collagen or a collagen‐based interpenetrating network (IPN) hydrogel, designed to reproduce the 3D structure of the colon and support co‐culture of epithelial and stromal cells under mechanical stimulation. The complexity of the colon‐on‐chip was designed to capture essential stromal–epithelial interactions of the intestinal niche and to account for mechanical forces acting on both epithelial and stromal cells. The IPN scaffold was adapted from a strategy recently described for unstructured vascular tissue engineering [25]. It combines collagen type I, which provides physiologically relevant cell‐adhesion sites, with PEGDA, a synthetic polymer that enhances mechanical strength and stability. PEGDA was selected for its high optical transparency, hydrophilicity, biocompatibility, and tunable mechanical properties [26]. Compared to pure collagen, the resulting composite hydrogel exhibits higher stiffness, enabling us to probe how matrix rigidity modulates cellular responses to cyclic stretching.

To construct the collagen‐PEGDA IPN scaffold, we first optimized photopolymerization parameters, including the photoinitiator type and concentration, irradiation time and intensity, to ensure maximal cell viability. Two photoinitiators were tested, Irgacure 2959 and lithium phenyl (2,4,6‐trimethylbenzoyl) phosphinate (LAP), on two cell types present in the ecosystem: (i) primary mouse intestinal fibroblasts (MIFs) embedded within the IPN and (ii) mouse intestinal organoids cultured on the scaffold. More precisely, fibroblasts (1.2 × 10^6^ /ml) were mixed with a 10 mg/mL collagen prepolymer solution, polymerized, and allowed fibroblasts to recover for 24 h. Subsequently, the PEGDA precursor solution (6 kDa at 11.7% w/v) containing the photoinitiator, was then allowed to diffuse for 2 h before UV photopolymerization. We found that photoinitiator concentrations exceeding 0.4% (w/v) resulted in massive cell death for both photoinitiators (Figure S1).

**FIGURE 1:**
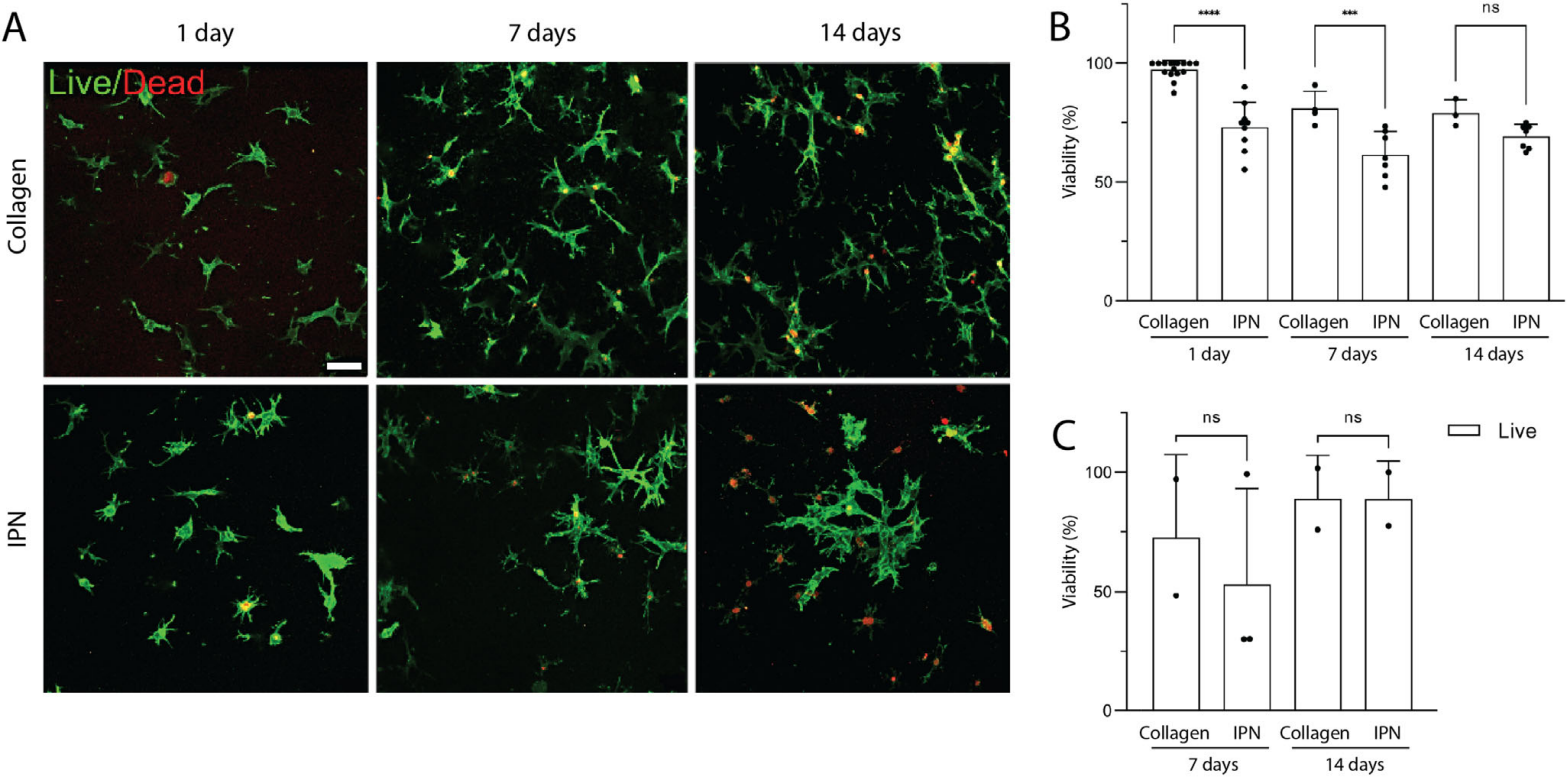
Cell survival in collagen and IPN scaffolds over long‐term culture. (A) Fibroblasts embedded in collagen (top panels) and IPN hydrogel (bottom panels) and cultured for 1, 7 and 14 days. Day 1 corresponds to one day after collagen polymerization. Fibroblasts are mixed with the collagen prepolymer solution prior to on‐chip injection and molding (with cylindrical crypt design), followed by the polymerization of collagen and the photopolymerization of PEGDA in the case of the IPN hydrogel. Live cells (green), the apoptotic nuclei (red). Scale bar, 50 µm. (B) Percentage of viable fibroblasts relative to the total number of cells in the IPN and collagen gels over time estimated with live‐dead assay.(C) Percentage of viable epithelial cells seeded atop of IPN and collagen gels over time relative to the total number of cells evaluated via Alamar blue test. Organoids viability is not measured at day 1 as organoids need 4 to 5 days to spread and cover the scaffold. Average total number of fibroblasts per z‐stack *n* = 22‐105, *N* = 2‐3 independent experiments. Mean ± SEM, One‐way ANOVA ^***^ *p*<0,0001; ^**^ *p*<0,001; ^*^ *p*<0,05.

As the absorbance of Irgacure 2959 is not optimal at 365 nm, for subsequent experiments, we only focused on LAP at a lower concentration, 0.2% (w/v), and used pure collagen hydrogel as a control. In these optimized conditions, at day 0 the fibroblasts were mixed with the collagen prepolymer before on‐chip injection and molding, followed by collagen polymerization and PEGDA photopolymerization in the case of the IPN. We next evaluated the ability of both scaffolds to sustain long‐term cell survival. Fibroblasts viability within the gels was monitored at 1, 7 and 14 days post‐encapsulation. We observed that fibroblasts retained their typical spindle morphology in both scaffolds (Figure 1A), with 70 ± 5.2% viability in IPN gels and 79 ± 5.7% in collagen after 14 days (Figure 1A, B). Similar experiments were performed with intestinal organoids seeded on the scaffold, except that day 1 measurement was not performed for organoids as they had not yet fully covered the hydrogel scaffold to form a confluent epithelium. After 14 days, the organoid viability was about 88 ± 15.9% on IPN's compared to 89 ± 18.2% on collagen scaffolds (Figure 1C).

Together, these results demonstrate that the optimized collagen–PEGDA IPN hydrogel preserves the viability and morphology of both stromal and epithelial cells, making it suitable for long‐term culture and subsequent mechanical stimulation experiments.

### 3.2 Cyclic Stretching Affects Collagen and Collagen‐PEGDA IPN Mechanical Properties

We next compared the mechanical properties of collagen I/PEGDA IPN with those of pure collagen I by performing first swelling measurements. The samples were let to swell in medium for 24 h. The medium was then removed and the samples gently blotted before to record the swollen weight. Finally, the samples were placed in an oven at 70°C for 48 h to dry them before reweighing. These measurements enabled us to determine the final concentration of both polymers in the hydrogel at equilibrium as explained in the M&M section. For pure collagen hydrogel, as expected, the final concentration of collagen I was at 10 mg/ml, i.e. 1 wt.%. For the IPN hydrogel, synthesized through a two‐step process involving a step of PEGDA diffusion, the final concentration of PEGDA within the IPN was estimated at 6.3 ± 4.1 wt.%.

**FIGURE 2:**
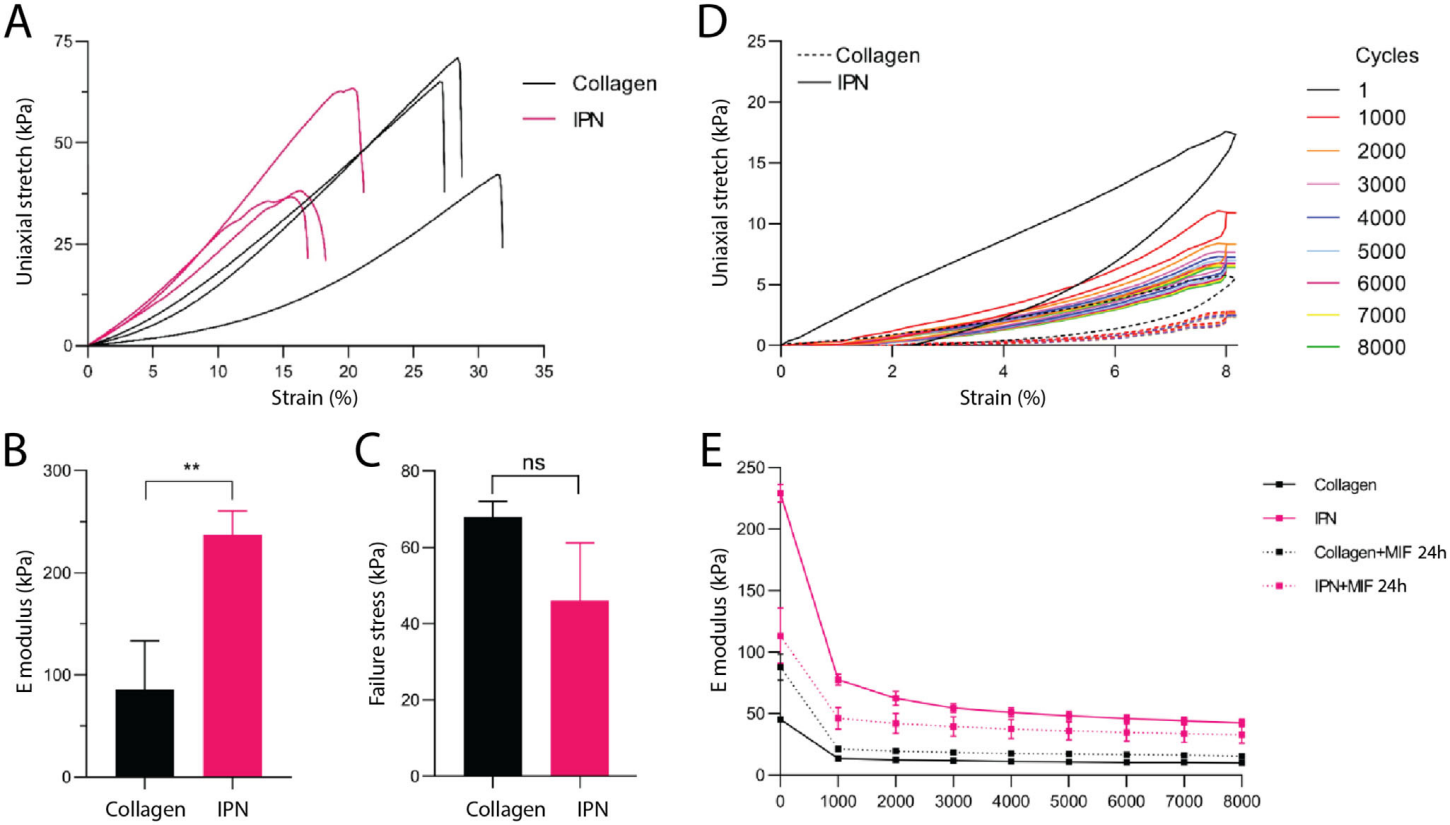
IPN mechanical properties characterization. (A) Stress–strain curves of collagen (black) and IPN gel (pink). (B) Tensile modulus (E) of collagen (black) and IPN gel (pink). (C) Failure stress of collagen (black) and IPN gel (pink). (D) Stress response of IPN (solid line) or collagen (dotted line) hydrogel to long‐term cycling constraints (8000 cycles) under 8% elongation at a frequency of 0.1 Hz. Every thousand cycles were represented in a different color. (E) Evolution of the Tensile modulus (E) of the IPN (pink line) and the collagen (black line) hydrogels over the cycles without (solid lines) and with embedded fibroblasts (dotted lines) cultured for one day within the gel prior stretching. For each condition, *n* = 3–6 independent samples were measured. Mean ± SEM, *t*‐test, * significantly different from the collagen 10 mg/mL hydrogel (p ≤ 0.01).

To probe the mechanical properties of these hydrogels, we used uniaxial tensile tests. Although this configuration does not fully replicate the complex peristaltic constraints in vivo*—*where both circular and longitudinal muscles contribute—it primarily mimics the strain generated by longitudinal muscle contraction. More importantly, it allows characterization of hydrogel mechanics under conditions similar to those applied later in the colon‐on‐chip system. To prevent hydrogel desiccation and maintain equilibrium chemical conditions (pH, ionic strength) like those of cell culture [27, 28] tensile tests were carried out immersed in a cell medium that was pH‐buffered. Specific jaws designed for gels were used to prevent gel slippage during testing (Figure S2A). We first performed tensile tests until rupture (Figure 2A). These tensile tests refer to an increase of the gel length L with respect to its reference state L_0_, so that ΔL > 0. The tensile behavior of both the collagen I and IPN hydrogels were non‐linear, the stress–strain curve exhibiting a J‐shaped typical of biological tissues [29, 30, 31] (Figure 2A). From the linear segment of these curves (Figure S2B), we estimated the Tensile's modulus (E) of each gel (Figure 2B), revealing that, as expected, the IPN has higher stiffness compared to the pure collagen hydrogel (237 ± 23.6 kPa vs. 85.7 ± 48 kPa, respectively). In addition, these tests provided a better understanding of the rupture properties of the gels, with pure collagen hydrogel exhibiting failure around 65 kPa, which is of the same order of magnitude as previously reported [29] (Figure 2C). The observation that collagen breaks more abruptly than the IPN (Figure 2A) could be attributed to a damping effect introduced by the inclusion of the PEGDA component.

Next, to assess gel behavior and durability under conditions mimicking in vivo peristalsis‐like motions, we performed fatigue tests applying a cyclic strain of 8% at a frequency of 0.1 Hz. These stretching parameters were determined based on the gut physiology [32] and on the parameters applied in existing in 2D‐like gut on chip models [7]. Notably, this strain limit is below the onset of damage mechanisms leading to failure [33]. The mechanical properties of both IPN and collagen hydrogels were evaluated over long‐term experiments; the gels being subjected to constant cyclic stretching over a high number of cycles (8000 cycles per experiment) (Figure 2D). In both conditions, the gel response to stress was not purely elastic and hydrogels exhibited viscoelastic characteristics, marked by a hysteresis behavior more pronounced during the first stretching cycle (Figure S2C), followed by cycles with a slight residual strain but almost closed loops especially for the IPN. The hydrogels underwent stress accommodation by network rearrangement during the first stretching cycles, as evidenced by a larger area under the curve for the first cycle compared to later ones (Figure S2C). The stiffness of the IPN decreased (by approximately 80%) until stabilizing at a plateau after 1000–2000 cycles of stretching. The control gel, composed solely of collagen I, displayed a similar trend, with a notable reduction in Tensile's modulus (also around 80%), leading to a plateau that was consistently lower than that observed for the collagen‐PEGDA IPN (≈10 kPa vs. ≈50 kPa) (Figure 2E, solid lines). Additionally, to further explore the effect of cyclic stretching on hydrogels, we compared the IPN's response under continuous cyclic stretching to that under intermittent stretching conditions, which involved 1 h of stretching followed by 2 h of rest, repeated four times for a total of 4 h of stretching. The results showed comparable patterns with no discernible impact of the relaxation period on gels’ stiffness (Figure S2D,E). These results suggest that most of the gel rearrangement occurs during the first cycles.

Finally, considering that fibroblasts can affect the ECM's mechanical properties by producing, degrading, and remodeling the ECM, we performed similar experiments with gels containing fibroblasts at the same density as used in the viability assay. Following one day in culture, a cycling experiment (8000 cycles, 8% strain, 0.1 Hz) was performed to assess changes in gel stiffness. We observed that the presence of fibroblasts reduced the initial stiffness of the IPN, indicating that the cells, as inherently softer than hydrogels, affected the overall mechanical response. For both types of hydrogels, we observed a decrease in stiffness with cyclic stretching similar to the experiment without fibroblasts, though the IPN gels maintained about twice the stiffness compared to the collagen (Figure 2E, dotted lines). This trend was consistent with the limited existing literature regarding the impact of cells on the mechanical properties of hydrogel [34]. Overall, this fine‐tuned IPN hydrogel fulfills the prerequisites for a stretchable 3D colon‐on‐chip application, providing enhanced mechanical properties over pure collagen and supporting the co‐culture of epithelial and stromal cells.

### 3.3 Development of a 3D Stretchable Gut on Chip

To reproduce the structural complexity of the colon and study the effect of stretching on intestinal cells, we next developed a 3D colon‐on‐a‐chip integrated with a custom uniaxial stretching device [24]. This device operates under standard cell culture conditions, accommodates two chips simultaneously and allows precise control of strain amplitude and frequency.

**FIGURE 3:**
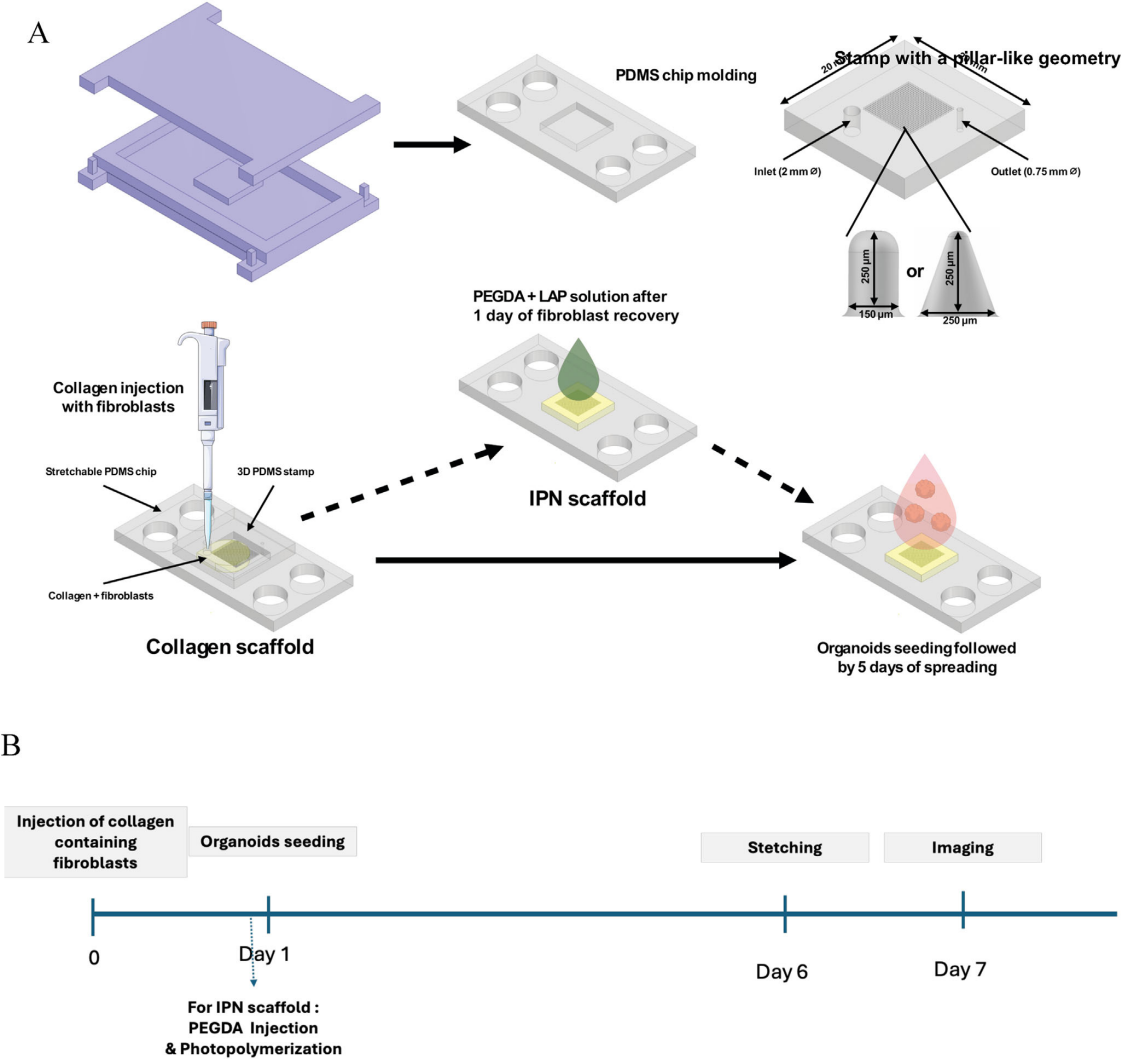
Colon‐on‐chip microfabrication. (A) Colon‐on‐chip microfabrication. (B) Timeline of the experimental workflow.

Inspired by the 2D device of Gérémie et al. [35], our chip is made of PDMS (Polydimethylsiloxane) contains a central chamber that hosts a 3D hydrogel scaffold and four peripheral holes for attachment to the stretching device (Figure 3; Figure S3). The inner surface of the chamber was functionalized with glutaraldehyde to promote covalent anchoring of collagen scaffold to the PDMS. We next fabricated a collagen‐based scaffold that mimics the 3D topography of the colon using a stamp with a pillar‐like geometry (28 × 28 crypts per device), which imprints an array of crypt‐like invaginations. The crypt dimensions were chosen to approximate physiological colonic crypts while remaining compatible with the resolution limits of 3D‐printed mold fabrication. During fabrication, the stamp was aligned and sealed atop the main chip chamber, and a collagen prepolymer solution containing fibroblasts was injected through the inlet port. After polymerization, the stamp was gently removed, leaving behind a stable 3D collagen scaffold with defined architecture (Figure S4). For the IPN‐based scaffold, following collagen polymerization and a 24‐h recovery period for the fibroblasts, a PEGDA solution was applied to the gel surface and allowed to diffuse for 2 h prior to photopolymerization. To mimic the basement membrane that separates the epithelium from the stroma, the scaffold was coated with laminin via physisorption. Finally, to reconstitute the intestinal epithelium, mouse intestinal epithelial organoids approximately 100 µm in diameter were seeded onto the 3D scaffold (Figure 3). Within five days, the epithelial cells expanded and formed a confluent monolayer that mirrored the crypt dimensions defined by the mold (Figure 3; Figure S3B).

### 3.4 Cyclic Stretching did not Significantly Increase Fibronectin Production by Fibroblasts

Fibroblasts are key producers of the ECM, notably fibronectin, which is crucial for cell adhesion, migration, and differentiation [5, 36] and they also modify the ECM's mechanical properties. The fatigue tests performed in presence of fibroblasts within the hydrogels showed that fibroblasts embedded in the gel altered its mechanical properties and its response to stretching (Figure 2E).

**FIGURE 4:**
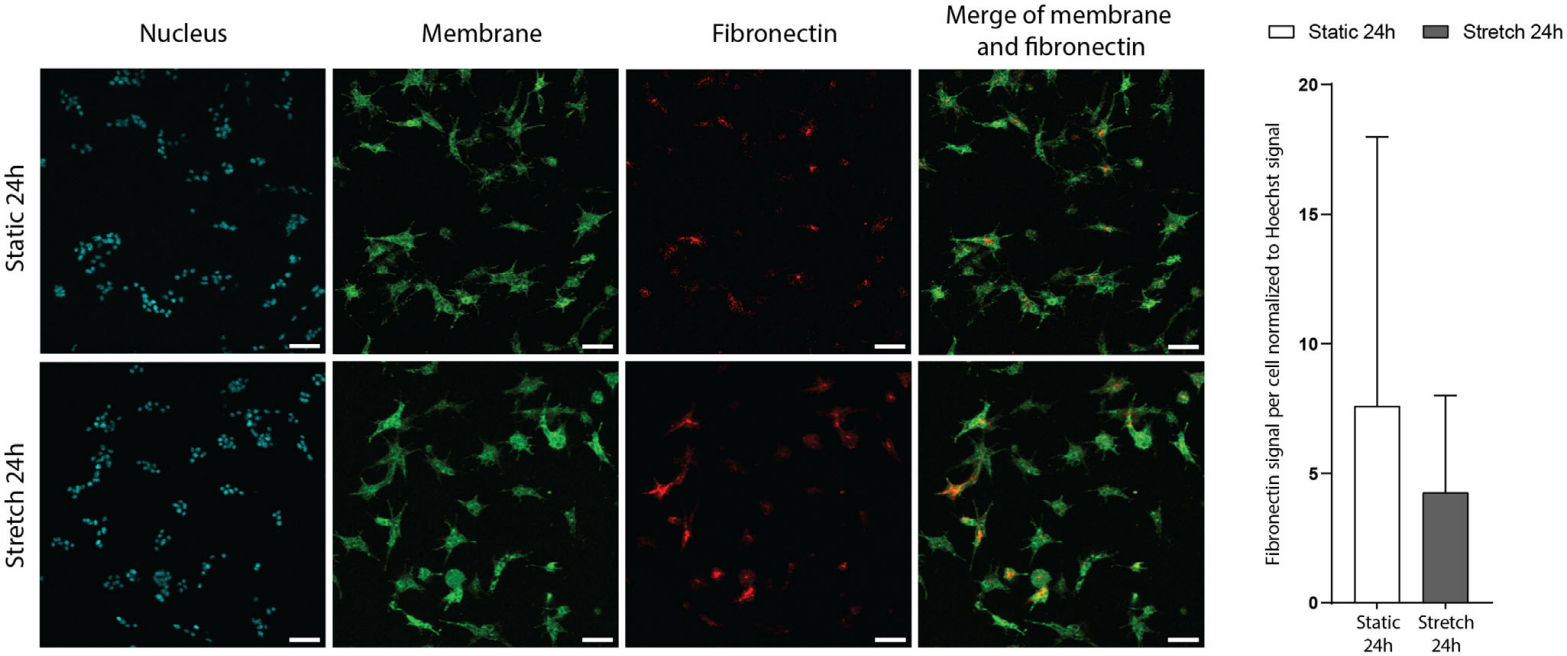
Comparison of fibronectin production under static or stretching conditions. (A) Fibroblasts at day 1 embedded in IPN gel in static (top panels) and stretched (bottom panels) conditions. Fibroblasts (membrane, green and nuclei, cyan) and fibronectin (magenta). Scale bar, 50 µm. (B) Quantification of fibronectin signal per cell normalized to Hoechst signal at day 1. *n* = 5–10 z‐stacks per conditions, *N* = 2 independent experiments.

Using our colon‐on‐chip model, we next investigated whether cyclic stretching impacts fibronectin production by fibroblasts within the IPN scaffold. To mimic intestinal peristalsis observed in vivo, we subjected the chip to cyclic stretching for 24 h at a physiologically relevant frequency of 0.1 Hz and an 8% strain amplitude [7, 37]. For comparison, we used control 3D scaffolds that remained static throughout the experiment. We quantified the amount of fibronectin produced by fibroblasts subjected to peristalsis‐like motion and compared it to static conditions for IPN scaffold. After one day of stretching, fibroblasts exhibited a modest, though not statistically significant, increase in fibronectin deposition relative to static conditions (Figure 4A, B). This suggests that 24 h of peristaltic stimulation does not significantly enhance ECM production.

### 3.5 Peristalsis‐Like Motion Enhances Apicobasal Polarity of Epithelial Cells

**FIGURE 5:**
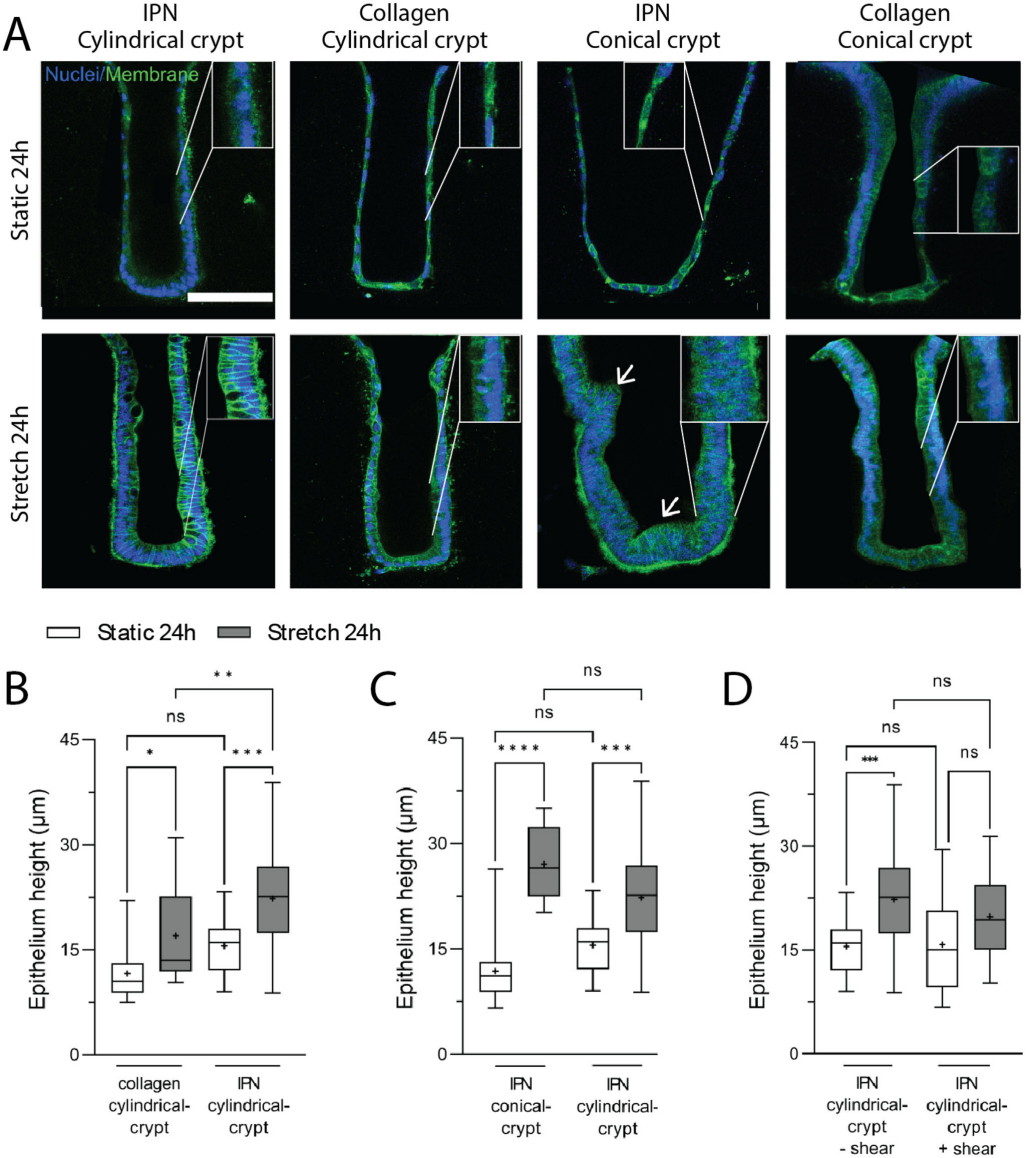
Cell polarization is improved with peristalsis‐like motion after 1day. (A) Representative longitudinal images of crypts after 24 h in static condition (top panels) and subjected to stretching (bottom panels) for, from left to right, IPN cylinder‐like crypt, collagen cylinder‐like crypt, IPN cone‐like crypt and IPN cylinder‐like crypt under shear stress. Nuclei are in blue and membranes in green. Scale bar, 100 µm. (B) Quantification of the epithelium height comparing 24 h of stretching vs. static conditions for collagen and IPN cylindrical‐crypts. (C) Quantification of the epithelium height comparing 24 h of stretching vs. static conditions for IPN cylindrical‐crypts and conical‐crypt. (D) Quantification of the epithelium height comparing 24 h of stretching vs. static conditions for IPN cylindrical‐crypts in the presence or absence of shear stress. n = 10‐42 crypts per conditions, N = 2 independent experiments. N does represent the number of independent experiments, and n represent the total number of crypts analyzed. Median ± SEM, the mean is symbolized by +, One‐way ANOVA ^***^ *p*<0,0001; ^**^ *p*<0,001; ^*^ *p*<0,05.

We further investigated the impact of cyclic stretching on the epithelium when cultivated on a 3D scaffold containing intestinal fibroblasts. We observed that mouse intestinal organoids, whether subjected to stretching or not, spread across the 3D IPN scaffold and colonized the crypts, forming a locally confluent epithelial monolayer. More notably, we found that just one day of cyclic stretching had a profound effect on the organization of the epithelial tissue. Under stretched conditions, epithelial cells within the crypts were densely packed, adopting a columnar shape with nuclei oriented perpendicularly to the basal side, as observed in vivo, while, in static conditions, most of the cells appeared flattened, almost squamous in shape and oriented circumferentially around the crypts (Figure S5A), aligning with the direction of maximal curvature. At the plateau region, the cells displayed a hexagonal shape at their apical side characteristic of the in vivo epithelial morphology (Figure S5B). The enhanced epithelial polarization under cyclic stretching was further confirmed by staining the epithelial cells for actin and NaK‐ATPase, an intestinal transport enzyme and polarization marker [38, 39]. F‐actin was found enriched at the apical side (Figure S5C) while NaK‐ATPase was localized laterally in the polarized IPN cylindrical‐like stretched crypt, while it was poorly expressed in the non‐polarized static conditions (Figure S5D). To further assess epithelium polarity, we measured the epithelium height across the entire crypts and observed over all the conditions a 1.6 ± 0.5 fold increase of the epithelial cell height upon stretching compared to static conditions (Figure 5A). This effect of stretching on epithelium polarization was next confirmed on extended period of stretching of 3 days (Figure S6A) and for different patterns of stretching frequency (Figure S6B).

We next examined if the epithelium response to stretching could depend on the hydrogel properties by comparing the colon‐on‐chip model made either of pure collagen or of IPN, both with fibroblasts embedded within the scaffold. As shown above, tensile modulus of the IPN was about two‐fold higher than pure collagen I, even when subjected to stretching and in the presence of fibroblasts (Figure 2E). We first compared the epithelial organization in static conditions for both hydrogels (Figure 5A). We observed that epithelial cells were rather flat on a collagen scaffold, with an average cell height of 11.6 ± 4 µm. In contrast, on the IPN scaffold, cells exhibited an intermediate state with a mean height of 15.5 ± 4.2 µm, and some cells achieved full polarization, especially at the bottom regions of the crypts (Figure 5A) (Figure S7A). When subjected to stretching, epithelial cells grown on the collagen scaffold change their shape and organization, exhibiting mostly a cuboidal shape and an increased mean cell height of 17 ± 7.1 µm. This can be compared to the IPN conditions where cells, in stretching conditions, are uniformly polarized and present a mean cell height of 22.3 ± 7 µm (Figure 5B). For both hydrogels, the peristaltic‐like motion induced a change in cell orientation with more polarized cells after stretching, this effect being more pronounced on the stiffer scaffold.

Once it was established that matrix stiffness did not affect epithelial polarization under either static or stretched conditions, the remainder of the study was conducted using a single hydrogel formulation. The IPN scaffold was selected due to its superior mechanical stability and robustness under mechanical stress, which is essential for reliably investigating the effects of curvature and fluid flow in subsequent experiments.

Epithelial cell behavior being known to be sensitive to tissue curvature, we exploited microfabrication to investigate the effect of crypt geometry on epithelial polarization and responses to mechanical stimulation. We first showed that no significant deformation of the structure was observed after 24 h of stretching (Figure S7B). We next fabricated two IPN scaffolds differing in shape and curvature. The first 3D scaffold can be approximated as a cylinder, presenting a maximum curvature of 0.006 µm^−1^. In these conditions, the Gaussian curvature, which is the product of the maximum and minimum curvature, can be estimated to zero. The second scaffold harbors crypts with a progressive increase in diameter, resulting in cone‐like crypts (Figure 3) with a curvature ranging from 0.004 to 0.02 µm^−1^. Similar to the cylinder shape, the cone exhibits a zero Gaussian curvature on almost all of its surface. Under static conditions, cells on the cone‐like crypt scaffold were flat, with a cell height of 11.8 ± 4.1 µm. This contrasts with the cylindrical crypt scaffolds, as described above, where some epithelial cells were polarized at the bottom of the crypt (Figure 5B; Figure S7A). This difference in cell polarization as a function of crypt geometry in static conditions confirmed previous observations in other contexts [40]. Upon stretching, cells fully polarized in both crypt geometries, with an average height of the epithelium around 22 µm in the cylindrical crypts and 28.6 ± 7.1 µm in the cone‐shaped crypts (Figure 5C). The heightened polarization observed in the cone‐shaped crypt condition can be attributed to the existence of multilayered regions (Figure 5A, white arrows). We also assessed the impact of a reduced crypt aspect ratio with crypts of 150 µm in diameter and 150 µm in depth, and a similar effect of stretching was observed in these conditions (Figure S7C). Overall, these results suggest that stretching forces could erase the curvature effect and rescue the cell apicobasal polarization.

In vivo, the colonic mucosa is not only subjected to stretching but also to shear stress as feces move through the lumen [41]. It has already been shown in vitro that intestinal cells are sensitive to fluid shear stress [6, 22, 42]. We next explored how the fluid shear stress, combined with stretching, impacts the epithelial cell morphology. For this purpose, we used the IPN scaffold with a cylinder geometry. The chips were exposed to a fluid shear stress of approximately 0.06 dyn/cm^2^, which is within a similar range to the shear stress applied in other gut‐on‐a‐chip models and that observed in vivo [21]. In the absence of stretching, the average height of epithelial cells was around 15.8 ± 6.7 µm, similar to the condition without shear stress (mean cell height: 15.5 ± 4.2 µm). Under stretching conditions, either with or without shear stress, cells fully polarized, with height of 20 µm ± 5.7 µm and about 22 µm respectively (Figure 5D). These results suggest that shear stress had a limited effect in this configuration, in contrast with what has been observed with 2D chip where the flow shear stress was shown to be a critical determinant of cell response [21]. In our 3D colon model, which mimics the in vivo architecture, the confined spaces of the crypts may shield cells from shear forces, explaining the lack of observed differences in polarization between shear and no‐shear conditions. We confirmed this assumption by finite element (COMSOL) simulations modeling the shear stress gradient within this 3D scaffold. These simulations showed that for a shear stress of 0.06 dyn/cm^2^ on the flat surface of the scaffold, the shear stress decreased drastically in the first tens of micrometers in depth, approximating to zero (Figure S7D).

Altogether, these experiments highlight the high level of experimental control provided by this colon‐on‐chip model, establishing it as a powerful platform to dissect the respective contributions of mechanical forces and geometry on cellular behavior. They further demonstrate that peristaltic motion profoundly enhances epithelial cell polarization, independently of shear stress, scaffold curvature, or stiffness.

### 3.6 Peristalsis‐Like Motion Enhances Cell Proliferation Without Altering Differentiation

**FIGURE 6:**
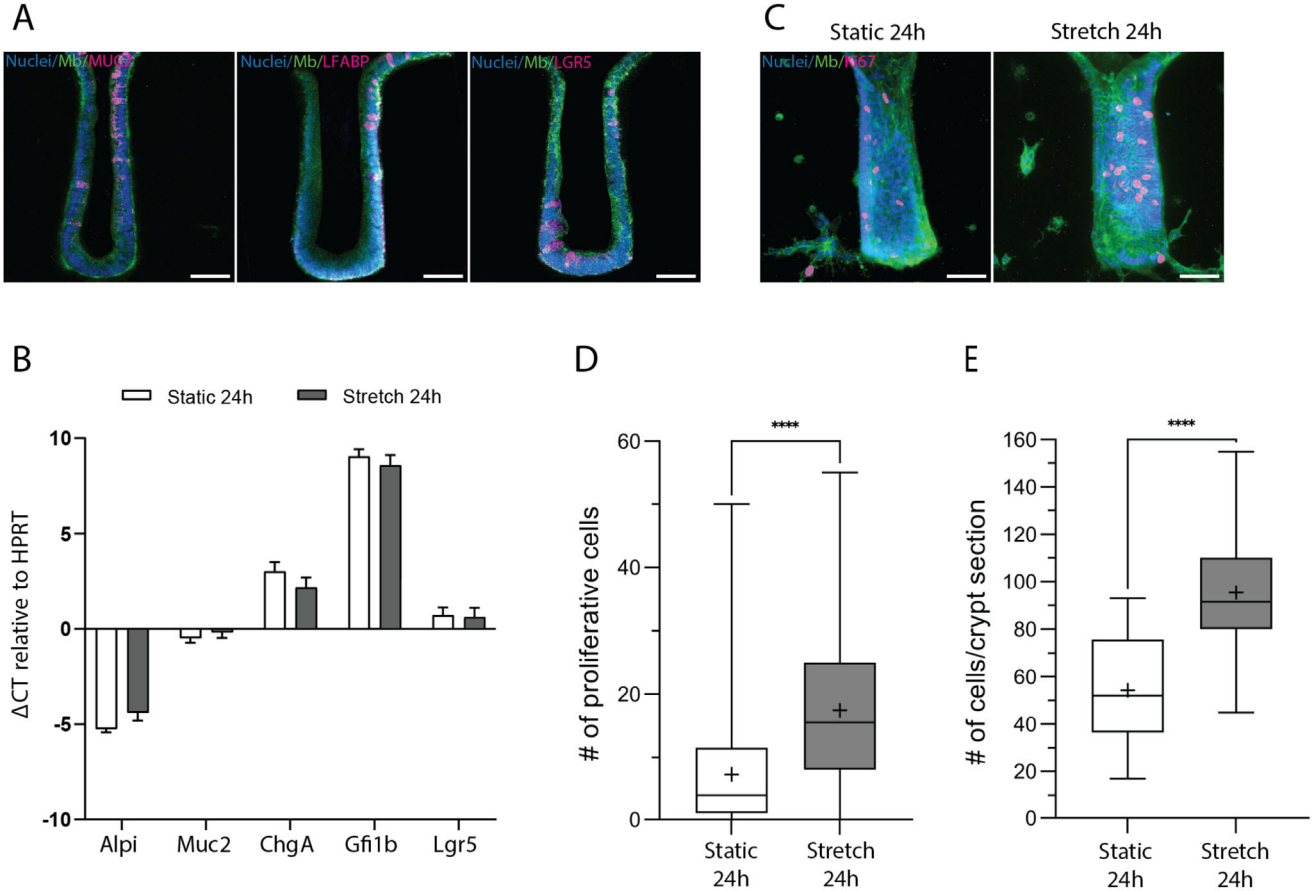
Epithelialization on IPN based cylindrical‐crypts scaffold after 1 day. (A) Goblet cells labeled with anti‐mucin2 antibodies, enterocytes labeled with anti‐LFABP antibodies and Lgr5 GFP labeled with anti‐GFP antibodies in the epithelial monolayer after 24 h of stretching with nuclei in blue, membranes in green and MUC2, LFABP and Lgr5 in magenta. Scale bar, 50 µm. (B) Fold change in the levels of transcripts from epithelial cells (enterocytes: alkaline phosphatase (ALPI), goblets: mucin 2 (MUC2), enteroendocrine cells: chromogranin A (ChgA), Tuft cells: Growth Factor Independent 1B Transcriptional Repressor (Gfi1b) and stem cells: leucine‐rich‐repeat‐ containing G‐protein‐coupled receptor 5 (Lgr5)) stretched on the colon on chip relative to the transcript levels in the static condition and assessed at 24 h. HPRT (hypoxanthine‐guanine phosphoribosyltransferase) is the housekeeping gene used to normalize RT‐qPCR data. Error bars correspond to SEM. With a 2‐way ANOVA test, no significant differences between stretched and static conditions (24 h) were observed. (C) Representative Z projection images of both static (top panel) and stretched (bottom panel) conditions. Scale bar 50 µm. Nuclei are in blue, membranes in green and Ki67 positive cells in magenta. (D) Quantification of the Number of Proliferative Cells Per Crypt. (E). Quantification of the number of cells per crypt section *n* = 113 crypts per conditions extracted from 5–10 chips, *N* = 2 independent experiments. Mean ± SEM, *t*‐test, ^***^ *p*<0,0001.

Finally, we explored the impact of cyclic stretching on epithelial cell differentiation and proliferation. Based on the previous observations, these experiments were conducted using the IPN scaffold with a cylindrical crypt geometry. In vivo, epithelial cells are compartmentalized with the stem cells localized at the bottom of the crypt and the differentiated cells at the upper part of the crypt. After 24 h of cyclic stretching, the main epithelial cell types—including goblet cells (MUC2^+^), enterocytes (LFABP^+^), and Lgr5^+^ stem cells—were all detected within the colon‐on‐chip epithelium (Figure 6A), confirming that differentiation occurred under these conditions. To assess weather mechanical stimulation, affect lineage specification, we retrieved the epithelial cells from the scaffold, extracted the RNA and performed RT‐qPCR analysis of lineage‐specific markers: *Alpi*, (enterocytes); *Muc2* (goblet cells), *Gfi1b* (tuft cells), and *Lgr5* (stem cells). Comparable expression levels were observed between stretched and static conditions (Figure 6B), indicating that cyclic stretching did not bias differentiation toward any specific lineage.

In contrast, proliferation was markedly affected by mechanical stimulation. Ki67 immunostaining revealed proliferative cells in both conditions, but their number increased 2.5‐folds following 24 h of cyclic stretching (Figure 6B,C). Of note, we also observed proliferating fibroblasts in the scaffold's bulk (Figure 6B, white arrows). Quantification of cell density within the crypts showed a nearly twofold increase in epithelial packing under stretched conditions (Figure 6D), consistent with enhanced proliferation and tissue compaction.

Altogether, these results showed that peristalsis‐like cyclic stretching significantly stimulates epithelial proliferation and increases cell density, without affecting differentiation into specific intestinal lineages.

## 4 Discussion

Intestinal cells are experiencing a wide range of forces in vivo. Here, we developed a new colon‐on‐chip that integrates a 3D scaffold made of hydrogel mimicking the colon architecture, populated with both epithelial and stromal cells, and capable of applying peristaltic‐like deformations. Owing to its high degree of mechanical and biological control, this model provides a powerful platform to dissect how mechanical cues affect the intestine cells. To our knowledge, this is the first system combining 3D architecture, epithelial–stromal co‐culture, and physiologically relevant cyclic stretching within a single device.

Because this system was specifically designed to mimic peristaltic motion, we first characterized the mechanical response of the hydrogel‐based scaffold to cyclic stretching. Fatigue tests comparing the IPN hydrogel to pure collagen gel revealed that the IPN exhibited a higher tensile modulus but, like collagen, underwent softening upon repeated stretching. This softening likely reflects internal fiber rearrangements within the polymer networks, leading to a new mechanical equilibrium as previously observed for collagen gels [29, 43].

We then investigated how embedded fibroblasts influence these properties. While it is well established that hydrogel mechanical properties such as stiffness, porosity, permeability affect cellular behavior [44]; the reciprocal impact of cells to their surrounding matrix has received far less attention. We found that the presence of fibroblasts induced a strong decrease in IPN stiffness, whereas pure collagen gels were mechanically similar regardless of cell inclusion. In both static and stretched conditions, pure collagen gels stabilized at a tensile modulus of ∼10 kPa after the initial loading cycles, consistent with fiber rearrangement and sliding within the collagen network [29]. Given that fibroblasts were encapsulated only 24 h before testing, their biochemical remodeling activity was likely minimal compared to the mechanical effects of stretching.

After around 3 h of stretching, both collagen and IPN hydrogels containing fibroblasts exhibited stiffness values of 10 to 50 kPa, within the physiological range reported for colonic tissue. Stewart et al. measured colon stiffness in kPa range across species range [45], although their data represent whole mucosal tissue, rather than the ECM alone. Recent work from our group demonstrated that stiffness of the basement membrane at the crypt bottom is around 10 kPa [46]. These differences highlight the challenges in providing the community with precise (and absolute) quantifications of in vivo mechanical properties of colon tissue. These measurements depend on the sample itself, its processing, and, most importantly on the characterization methods used, which can probe the tissue at varying scales.

We next developed a microfabrication workflow to construct a colon‐on‐chip model that combines key features of the organ: the colon crypt geometry, the co‐culture of fibroblasts and epithelial cells derived from organoids and the application of in vivo‐like mechanical cues, namely shear stress and peristaltic motion. To our knowledge, this is the first system combining these features within a single, controllable platform. In static conditions, epithelial cells were flattened, lacked the apicobasal polarity and aligned circumferentially along the crypts. This organization is consistent with previous observations showing that epithelial cells cultured on curved surfaces preferentially align along the direction of maximal curvature [47, 48, 49]. However, after one day of cyclic stretching mimicking in vivo peristaltic contractions, the epithelium underwent a profound reorganization: cells became densely packed, elongated, and displayed clear apicobasal polarity. This transition was accompanied by a marked increase in epithelial proliferation and cell density, indicating that mechanical stimulation promotes tissue compaction and maturation. Similar correlations between confinement, crowding, and epithelial organization have been reported by Lutolf and colleagues in microstructured cavities [40], supporting the idea that cyclic stretching enhances proliferation and tissue polarization through mechanical crowding. Surprisingly, despite the elevated proliferation rate the stem cell compartment did not expand after 24 h of cyclic stretching. This observation contrasts with the findings of Meng et al. [33], who reported expansion of the stem cell domain under stretching. However, their experiments were performed over longer durations and in a much softer matrix composed of Matrigel and collagen I, whereas our IPN scaffold exhibits a higher stiffness. Given that moderate stiffness favors to stem cell maintenance [50], the absence of stem cell expansion in our study reflects both the shorter timescale and the distinct mechanical properties of the matrix. Furthermore, our data suggest that peristaltic motion primarily promotes proliferation and tissue polarization without altering differentiation balance. Beyond the effect of stretching, our colon‐on‐chip enables the independent assessment of other microenvironmental cues—such as curvature, stiffness and shear stress—that are known to influence cell proliferation [51], differentiation [52] or migration [53]. However, so far, their effect is mostly studied individually. Our findings showed that peristalsis‐like motion has a dominant influence on epithelial organization, inducing polarization independently of scaffold curvature or stiffness. Moreover, in contrast to observations from 2D systems, where flow‐induced shear stress is a key regulator of epithelial behavior, we found that in our 3D biomimetic crypts, the contribution of shear stress is negligible compared to that of peristaltic strain.

Apicobasal polarity is a hallmark of epithelia and constitutes an important aspect of the physiology of an organ physiology. It ensures directional transport of ions, nutrients and signaling molecules, thereby maintaining tissue homeostasis and barrier function [38]. It helps define the cell shape and height and is characterized by a well‐organized distribution of the different cell‐cell junctions, such as the tight and adherens junctions along the vertical axis of the cell [54, 55]. The cell‐cell junctions and cell‐ECM interactions are mechanosensitive, intimately linked to the cytoskeleton that is instrumental for force transduction [56, 57, 58]. Ion channels are also stretch‐inducible and in consequence can be considered as good candidates for mechanotransduction [59, 60]. The establishment of apicobasal polarity is widely known to rely on three protein complexes that are evolutionarily conserved: Par, Scribble, and Crumbs. However, recent investigations focused on the Drosophila midgut have unveiled a distinctive pattern regarding epithelial polarity, setting it apart from other Drosophila epithelia where polarity factors cited above delineate the apical (Crumbs), junctional (Par‐3), and basolateral (Scrib) domains [61]. In mammalian epithelia, Crb3 governs tight junction formation in MDCK cells [62], yet its impact differs in the murine intestine, where it governs apical organization instead. This underscores the potential variability in polarity mechanisms within the gut and implies a differential regulation process of polarity that might differ between cell types and from 2D to in vivo 3D environment. To date, no comprehensive investigations have explored the factors orchestrating polarity within the mammalian intestine [63]. All these cited factors could be potential actors and thus targets to determine the main signaling pathway driving the stretch‐induced apicobasal polarization. Our colon‐on‐chip model offers a powerful platform to dissect these mechanisms, as it reproduces the mechanical, geometric, and cellular complexity of the native tissue while allowing precise perturbation of physical and biochemical parameters. Future iterations of this system, incorporating immune or stromal components, will make it possible to investigate how extrinsic signals and mechanical dynamics converge to regulate epithelial polarity under both physiological and pathological conditions.

## 5 Conclusion

This work represents a significant advancement in the field of organ‐on‐chip as it provides the community with a colon model of enhanced physiological relevance. By integrating cyclic stretching within a 3D framework encompassing both epithelial and stromal compartments, we have developed a unique model capable of dissecting the role of scaffold properties and external forces on intestinal cells. Here we performed a comprehensive study of the interplay between mechanical and geometrical cues and the cell morphology, proliferation and differentiation. Our approach not only provides a more faithful representation of the in vivo gut environment but also offers insights into the complex interactions driving physiological processes. This model can, in the future, be further extended to pathological conditions such as colorectal cancer on chip or inflammatory bowel disease. Our work offers a new perspective on gut‐on‐chip systems with a versatile and easy‐to‐handle model for which several parameters can be tuned at will to unravel the complex dynamics between biophysical cues and cellular behavior in physiological or pathophysiological contexts.

## Author Contributions

S.D. and D.M.V. conceived the study. M.B.D. developed the procedure to stiffen collagen, optimized fibroblast survival during the IPN synthesis, designed the molds, performed the experiments and immunostaining, image acquisition and analysis. L.C. participated to on chip experiments and revision experiments. J.C. designed the fibroblast extraction methods and performed their genetic modifications. R.B. provided decisive support for organoids and fibroblasts culture. L.G. and G.G. set up the stretching platform. J.B. and A.M. provided decisive support with their expertise in mechanical testing and analysis. M.B.D., S.D. and D.M.V. wrote the manuscript with input from all authors. D.M.V. and S.D. supervised the project.

## Conflicts of Interest

The authors declare no conflicts of interest.

## Supporting information




**Supporting File**: adhm70762‐sup‐0001‐SuppMat.docx.

## Data Availability

The data that support the findings of this study are available from the corresponding author upon reasonable request.
