# Supplementary material for: A New 3D Colon on a Chip to Decipher the Influence of Mechanical Forces on the Physiological Cellular Ecosystem
Source: Adv Healthc Mater. 2026 Jan 24;15(17):e05483. doi: 10.1002/adhm.202505483 (PMC13175293; doi:10.1002/adhm.202505483)

**Supplementary**

**Figure S1. MIF survival in other IPN formulations.** Representative images of embedded MIF in IPN photocrosslinked with Irgacure 2959 (I) 0.5% w/v (top panels) or LAP 0.4% w/v (bottom panels) over time. The apoptotic nuclei are stained in red and the cell bodies are in green. Scale bar, 50µm. Percentage of living (white) and dead (dark gray) MIF over time for each IPN. n=3-9 z-stacks for each time point and condition, N=2 independent experiments. Mean ± SEM.


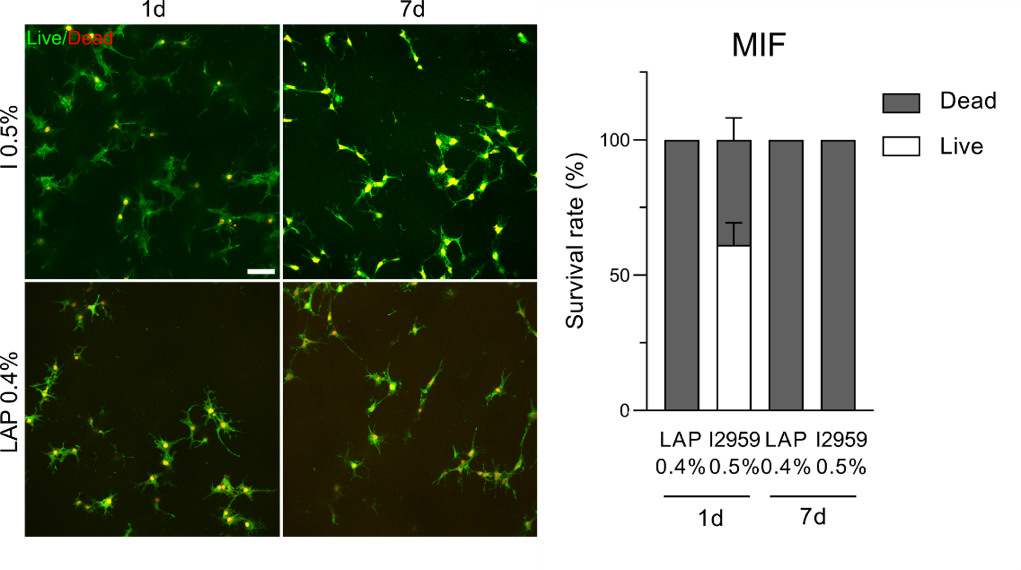


**Figure S2. Mechanical properties characterization.** Uniaxial traction experimental set up on an Instron (A). Tensile modulus extraction from the linear region of the stress-strain curves of collagen 10 mg/ml (black) and IPN gel (pink) (B). Normalized stress response of the cycles (8%, 0.1Hz) of IPN (solid line) and collagen (dotted line) hydrogel (C). Mean stress response of IPN (solid line) or collagen (hashed line) hydrogel to long term cycling constraints (8000 cycles) with two hours of rest after each 2000 cycles under 8% elongation at a frequency of 0.1 Hz. Every thousand cycles are represented in a different color (D). Evolution of the tensile modulus of the IPN (pink line) and the collagen (black line) hydrogels over the cycles (E). For each condition, n = 3–6 independent samples were measured.

**
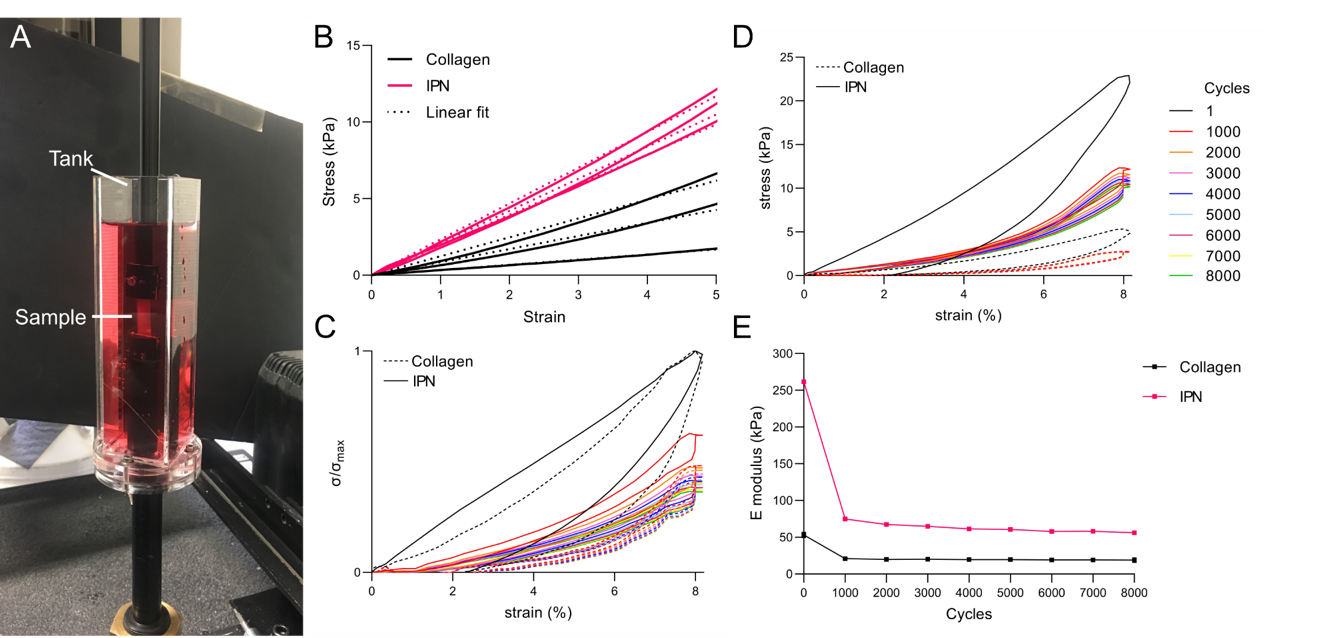
**

**Figure S3. Stretchable 3D colon-on-chip device**

1. Colon-on-chip design. The central chamber contains an array of 28x28 crypts and is surrounded by 4 holes to plug the chip on the stretching device, cross-section. Top panel: top view of the design. Bottom panel: cross-section of the chip design
2. Picture of the 3D colon-on-chip devices and picture of the epithelial monolayer over the IPN crypt-like scaffold 5 days after organoids seeding, in the presence of mouse intestinal fibroblasts. Nuclei are in blue and membranes in green.

**
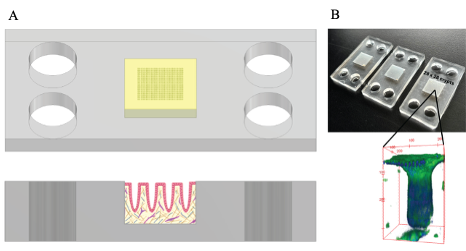
**

**FigS4. Molding characterization.** Dimensions of the theoretical cylinder-like crypt mold compared to the experimental IPN cylinder-like crypt scaffold after organoids spread and colonized the structure. n=17 crypts, N=2 independent experiments. Mean ± SEM.


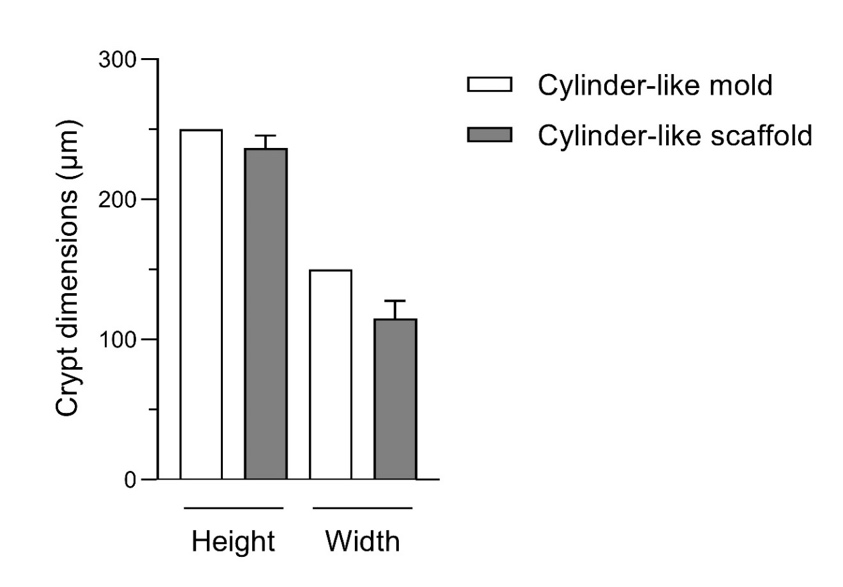


**Figure S5. Polarization characterization.** Cross-sections of IPN cylinder-like crypts in stretch and static condition at 1d. Scale bar, 100 µm (A). Top view of the plateau region between crypts on 1d stretch IPN based scaffold. Scale bar, 10µm (B). Zoom of an IPN cylindrical crypt stretched for 1d and stained for actin in green. (Phalloidin staining) Scale bar, 10µm (C). Representative images of NaKATPase staining in yellow of both 1d static non polarized (left) and 1d stretch polarized (right) cylinder-like crypts. Scale bar, 100µm (D).


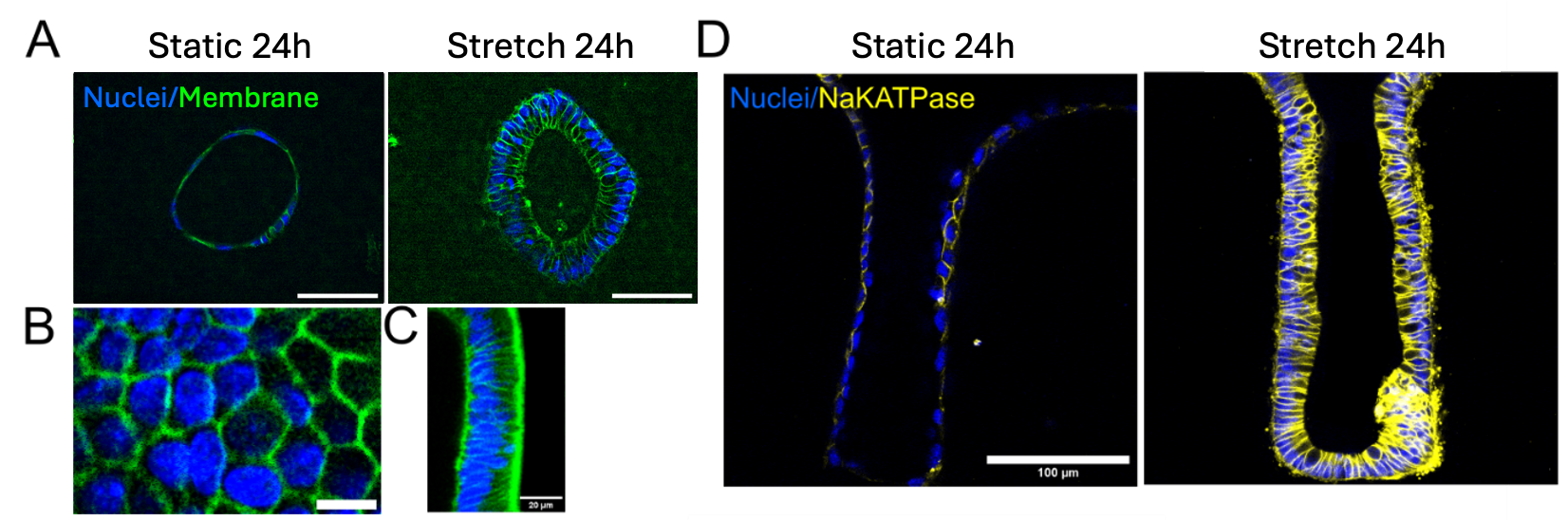


**Figure S6. Impact of extended stretching time and frequency pattern on cell polarization for IPN-based scaffold and cylindrical crypts**

(A). Quantification of the epithelium height for 72h of stretching (grey box) or 72h in static conditions (white box)

(B). Quantification of the epithelium height for different stretching conditions (24h)

(i) 24 static

(ii) 24h continuous stretch: frequency: 0.1 Hz, strain: 8%

(iii) 24h frequency noise stretch: frequency: 0.05-0.25Hz, strain: 0.8

(iv) 24h resting time stretch: frequency:0.1Hz, Strain: 8%, resting time 5 min, stretching time 1h.


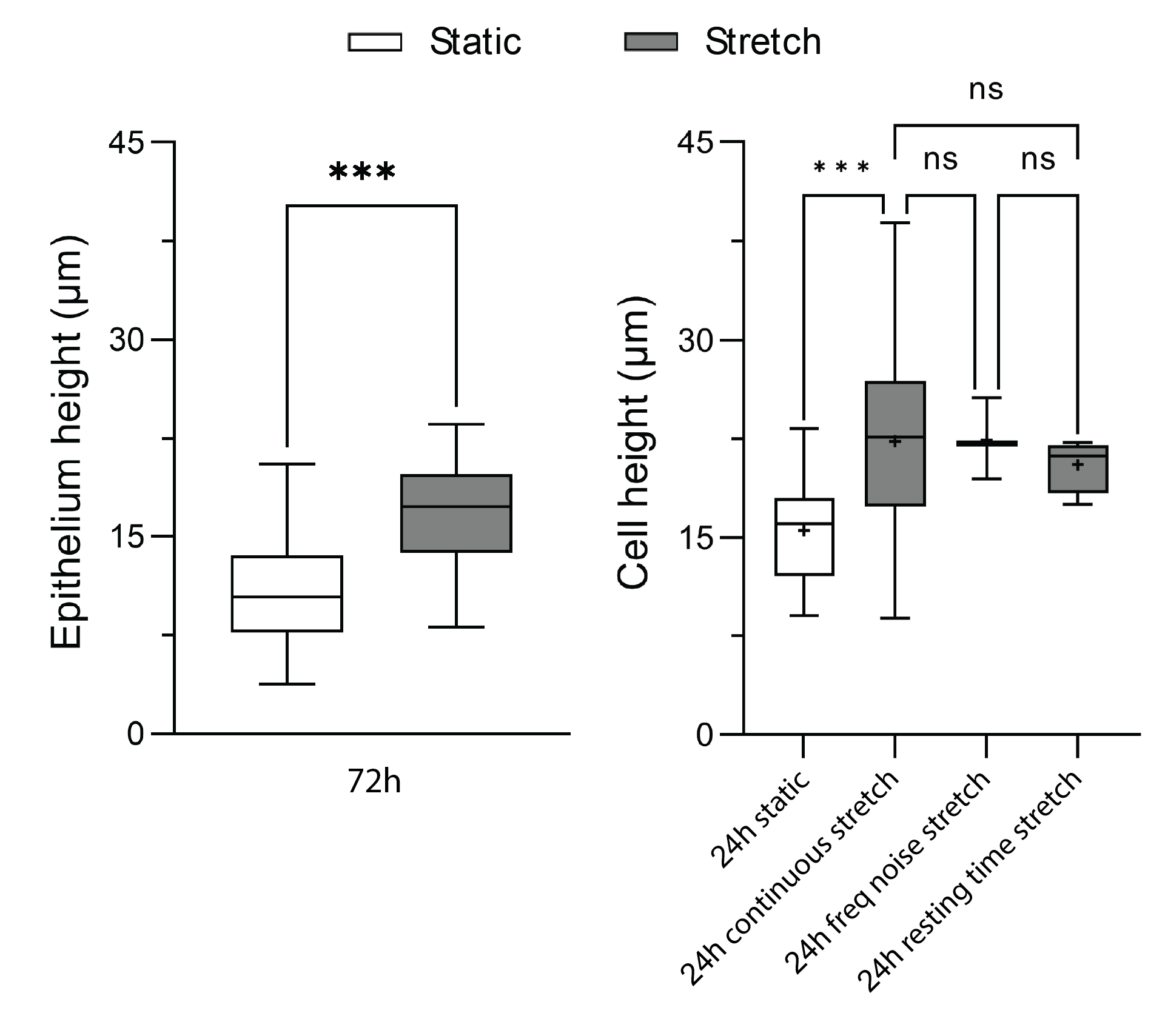


**Figure S7. Polarization as function of the cell positioning and shear stress.** (A). Quantification of the epithelium height per condition depending on the region of the crypt (B). Dimensions of the cylinder-like crypt scaffold in static and stretched conditions at day 1 for IPN scaffolds (C). Quantification of the epithelium height comparing 1d of stretching vs static conditions for IPN cylindrical-crypts with a reduced aspect ratio (AR) (150µm x 150µm) (D). Heat map (left) of the channel with the crypts in 2D. The maximum speed (red) is 2.10^-4^ m/s. Line graph (right) with the speed on the y-axis as a function of distance from the base to the bottom of the crypt, as indicated by the yellow arrow in the heat map, on the x-axis (E). In images, nuclei are stained in blue, membranes in green.


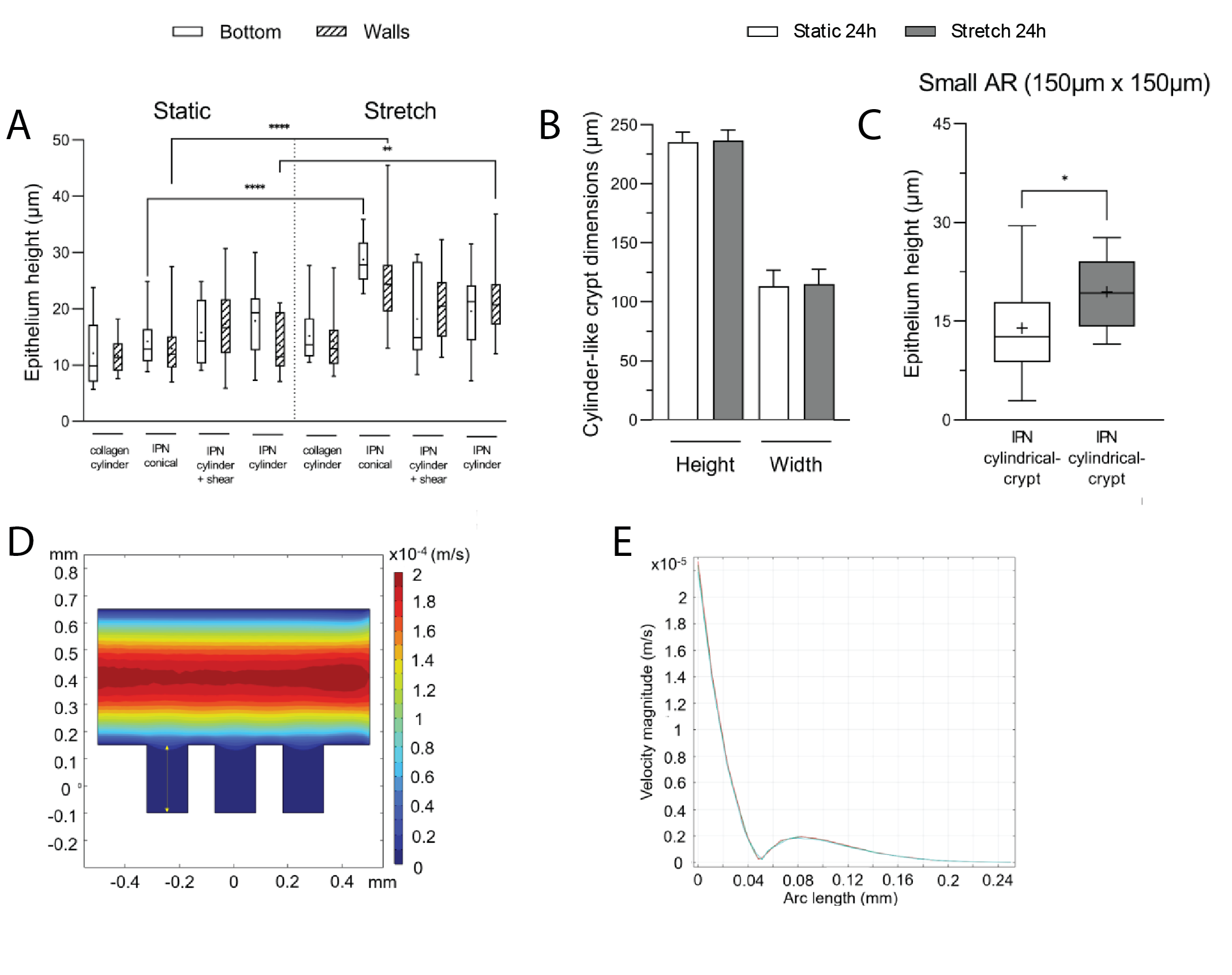

Supplement: Supplementary file 1 — Supporting File: adhm70762‐sup‐0001‐SuppMat.docx. [file ADHM-15-0-s001.docx]
